# Bifidogenic Effect of Human Milk Oligosaccharides on Pediatric IBD Fecal Microbiota

**DOI:** 10.3390/microorganisms12101977

**Published:** 2024-09-30

**Authors:** Nize Otaru, Danica Bajic, Pieter Van den Abbeele, Saskia Vande Velde, Stephanie Van Biervliet, Robert E. Steinert, Ateequr Rehman

**Affiliations:** 1 Health, Nutrition & Care (HNC), DSM-Firmenich, 4303 Kaiseraugst, Switzerland; 2 Cryptobiotix SA, Technologiepark-Zwijnaarde 82, 9052 Ghent, Belgium; 3 Pediatric Gastroenterology and Nutrition, Ghent University Hospital, Corneel Heymanslaan 10, 9000 Ghent, Belgium

**Keywords:** HMOs, IBD, CD, children, gut microbiota, bifidobacteria, 2′FL

## Abstract

The prevalence of pediatric inflammatory bowel disease (pIBD) has been increasing over the last two decades. Yet, treatment strategies are still limited, in part due to the multifactorial nature of the disease and the complex interplay between genetic, environmental, dietary, immune, and gut microbial factors in its etiology. With their direct and indirect anti-inflammatory properties, human milk oligosaccharides (HMOs) are a promising treatment and management strategy for IBD. However, to date there are no insights into how HMOs may affect pIBD microbiota. Here, we compared the effects of 2′fucosyllactose (2′FL), difucosyllactose (DFL), 3′sialyllactose (3′SL), and blends thereof with fructooligosaccharide (FOS) on microbiota functionality (short- and branched-chain fatty acids, pH, and gas production) and composition (quantitative shallow shotgun sequencing) using fecal material from eight different pediatric Crohn’s disease patients inoculated in the SIFR^®^ technology. In general, all HMO treatments significantly increased total short-chain fatty acid production when compared with FOS, despite equal gas production. We found that 2′FL, either alone or in combination with DFL and 3′SL, exhibited a strong acetogenic and propiogenic effect, and 3′SL an acetogenic effect that surpassed the effects observed with FOS. No differences in overall community diversity between HMO- and FOS-treated pIBD microbiota were observed. There was, however, a stronger bifidogenic effect of 2′FL, 3′SL, 2′FL/DFL, and 2′FL/DFL + 3′SL when compared with FOS. In general, 3′SL and HMO blends enriched a broader species profile, including taxa with potentially anti-inflammatory properties, such as *Faecalibacterium prausnitzii* and *Blautia* species. This study suggests HMOs as a promising strategy to beneficially alter the gut microbial profile in pIBD.

## 1. Introduction

Inflammatory bowel disease (IBD) is characterized by gastrointestinal (GI) inflammation, including periods of remission and relapse. Symptoms include both intestinal and extraintestinal features, including diarrhea, abdominal pain, fever, and weight loss [1,2]. Ulcerative colitis (UC) and Crohn’s disease (CD) are two subtypes of IBD. In UC, the inflammation is continuous and restricted to the colon epithelial lining, whereas in CD, it is transmural, irregular, and can occur anywhere in the GI tract [2]. Pediatric IBD (pIBD) patients often exhibit a more extensive and active disease when compared with adult patients [3]. Prevalence of pIBD has been increasing over the last two decades with the highest values seen in Northern Europe (75.0 per 100,000 persons) and North America (28.3 to 63.6 per 1,000,000 persons) [4]. Yet, etiology is not fully elucidated, but involves a complex interplay between genetic, dietary, environmental, immune, and gut microbial factors leading to an inappropriate immune response [5]. Treatment strategies for pIBD include pharmacotherapy, exclusive enteral nutrition, and gut microbiota-targeted interventions [6,7]. The gut microbiota is well known as being at the interface of gut epithelial inflammation with both bacterial surface structures (e.g., lipopolysaccharides and cell surface β-glucan/galactan) and bacterial metabolites (e.g., short-chain fatty acids (SCFAs)) playing a key role in the inflammatory process [8,9]. An imbalance in gut microbial composition and functionality, often referred to as dysbiosis, has been associated with pIBD [10,11]. Gut microbial community shifts often include a decrease in abundance of *Bifidobacterium* spp. and the phylum Firmicutes [11,12]. Acetate has been reported to be generally decreased in pIBD patients, while heterogeneous profiles were observed regarding butyrate and propionate [11]. Thus, biotic interventions exhibiting anti-inflammatory properties both through direct (modulation of host cells) and indirect (modulation of microbiota) effects are promising candidates in treating IBD patients.

Human milk oligosaccharides (HMOs) are the third-most abundant component of breast milk, after lactose and lipids [13]. They are oligosaccharides consisting of lactose and any combination of galactose, fucose, N-acetylneuraminic acid, and N-acetyglucosamine [14]. Thus, neutral fucosylated (e.g., 2′Fucosyllactose (2′FL)), neutral non-fucosylated, and sialylated (e.g., 3′Sialyllactose (3′SL)) HMOs can be distinguished [13]. The beneficial effect of HMOs in shaping the infant gut microbiota has been investigated extensively; moreover, breastfed infants are thought to have a reduced risk of developing IBD later in life [13,15]. More recently, the health-promoting properties of HMOs beyond infancy have also gained traction, with studies demonstrating a beneficial effect of HMOs in a school-aged pIBD patient or in healthy adults [16,17]. The underlying mechanisms are believed to involve fermentation of HMOs by resident microbiota leading to the production of SCFAs and other beneficial metabolites, as well as a direct interaction of HMOs with host immune and epithelial cells [14]. With regards to IBD, HMOs have been shown to exhibit anti-inflammatory properties on ex vivo intestinal tissue [18], to alleviate colitis in mouse IBD models [19,20], and to improve systemic symptoms in a pilot study with adult UC patients [21]. An alleviation of IBD symptoms in response to HMOs combined with probiotic *Lactobacillus* and *Bifidobacterium* strains was also found in a recent case study involving a pediatric patient [16]. To date, there are no studies available that have comprehensively investigated the isolated effects of HMOs on pIBD gut microbiota. Therefore, we aimed to assess the effects of a range of HMOs on fecal microbiota of pIBD patients and compare them against the well-established prebiotic fructooligosaccharide (FOS).

## 2. Materials and Methods

### 2.1. Donor Sourcing and Fecal Sample Collection

Eight pIBD donors were sourced at the University Hospital Ghent. Ethical approval for utilizing fecal samples, as described in this study, was given by the ethical committee of the University Hospital Ghent (reference number BC-09977). Informed consent was given by individuals directly. Inclusion criteria were: (i) being between six and eighteen years of age; (ii) suffering from CD with active disease state, including fecal calprotectin levels of >300 mg/kg (measured up to three months before providing a stool specimen, given that CD therapy did not change during this period); and (iii) being under treatment with anti-tumor necrosis factor α agents (infliximab monotherapy or infliximab + azathioprine combo therapy). Exclusion criteria were use of (i) antibiotics and (ii) probiotics/prebiotics in the past 90 days before providing a stool specimen.

### 2.2. Test Products and Ex Vivo Fermentation

To evaluate the effect of HMOs on pIBD microbiota, two different HMOs, namely 2′FL (DSM GlyCare^TM^ 2FL 9000, DSM-Firmenich, Esbjerg, Denmark) and 3′SL (DSM GlyCareTM 3SL 9001, DSM-Firmenich), and two different HMO-blends, namely 2′FL/Difucosyllactose (DFL; GlyCare™ 2FL/DFL 8001, DSM-Firmenich) and 2′FL/DFL + 3′SL (80% 2FL/DFL + 20% 3′SL (*w*/*w*)), were tested. All treatments were compared with a non-substrate control (NSC) (containing only nutritional medium blend and fecal inoculum) and the well-established prebiotic FOS (Sigma F8052, Sigma-Aldrich, Overijse, Belgium).

Effects of test products on pIBD microbiota were assessed using the SIFR^®^ technology, processing bioreactors in parallel using a bioreactor management device (Cryptobiotix, Ghent, Belgium). Bioreactors were filled with nutritional medium blend (M017 Cryptobiotix, Ghent, Belgium), fecal inoculum, and test product, resulting in a 5 mL end-volume. All test products were included at a final concentration of 5 g/L. After being rendered anaerobic, bioreactors were incubated at 37 °C under continuous agitation at 140 rpm for 24 h (MaxQ 6000, Thermo Scientific, Thermo Fisher Scientific, Merelbeke, Belgium). Subsequently, gas pressure was measured in the headspace while pH was measured in the liquid sample using a pH electrode (Hannah Instruments Edge HI2002, Temse, Belgium). Additionally, samples were taken for analysis of SCFAs, branched-chain fatty acids (BCFAs), and microbial composition. 

### 2.3. Evaluation of SCFA and BCFA Profiles

SCFAs and BCFAs were assessed as described previously [22]. In short, 2 mL of a 1:3 (*v*/*v*) diluted sample in distilled water were mixed with 0.5 mL of 48% sulfuric acid. Subsequently, the mixture was homogenized with an excess of sodium chloride, 2 mL of diethyl ether, and 0.2 mL of 2-methylhexanoic acid as internal standard. After separation, diethyl ether extracts were analyzed via gas chromatography coupled to a flame ionization detector (Trace 1300, Thermo Fisher Scientific, Merelbeke, Belgium). Total SCFA levels were defined as the sum of acetate, propionate, butyrate, and valerate. BCFAs were defined as the sum of isobutyrate, isovalerate, and isocaproate. 

### 2.4. Microbial Compositional Analyses

Bacterial cells were obtained by centrifugation of 1 mL of fermentation effluent for 5 min at 9000× *g*. Subsequently, total DNA was extracted using the SPINeasy DNA Kit for Soil (MP Biomedicals, Eschwege, Germany), according to the manufacturer’s instructions. DNA libraries were prepared and sequenced as previously described [23]. In short, fragmentation of genomic DNA was carried out with Illumina Nextera XT fragmentation enzyme. Library preparation was performed using the Nextera XT DNA Library Preparation Kit (Illumina, San Diego, CA, USA) and IDT Unique Dual Indexes with 1 ng of DNA. Sequencing was performed on an Illumina. 

Nextseq 2000 platform 2 × 150 bp FASTQ files were analyzed for taxonomic profiles using Kraken (v2.1.1) with confidence set to 0.1 and UHGG (v2.01) as the reference database, supplemented with the human genome (to account for host contamination). 

### 2.5. Evaluation of Bacterial Cell Density

Total cell counts were assessed via flow cytometry. In short, fermentation effluent was diluted in anaerobic phosphate-buffered saline and stained with SYTO 16 at a final concentration of 1 µM. Samples were analyzed using a BD FACS Verse flow cytometer (BD, Erembodegem, Belgium). Data analysis was performed using FlowJo (v 10.8.1). 

### 2.6. Data Analyses and Visualization

Data and statistical analyses were carried out in R (v4.2.2; www.r-project.org). Principle component analysis (PCA) was performed on relative and centered abundances at family level using the package FactoMineR (v2.11) [24]. Alpha diversity analyses (observed species and Shannon diversity index) were performed on data rarefied to even sequencing depth, including the 621 most abundant species (abundance ≥ 0.1% in one sample) covering 96.5% of the total community using the package phyloseq (v1.40.0). For differential abundance calculations and regularized canonical correlation analysis (rCCA), relative abundance counts were transformed to quantitative data via correction, with total cell counts for each sample (cells/mL). Differential abundance analysis at the phylum level was performed, including the 621 most abundant species (species with at least 0.1% abundance in at least one sample). In-depth differential abundance analysis at the species level was performed on the top 150 most abundant species, covering 82.9% of the total community, using log_10_-transformed data. Values below the limit of quantification (LOQ) were considered equal to the LOQ, as previously described [25]. To capture treatment effects on taxa that were not present in all donors, non-significantly but consistently increased species for all donors where they were detected (with a minimum of 4 donors) were evaluated. The rCCA was performed on absolute abundances at the species level (including the 150 most abundant species) using the package mixOmics (v6.28.0) [26], including the shrinkage approach for regularization.

Evaluation of differences between the NSC and treatments and FOS and treatments was performed using repeated measures analysis of variance (ANOVA) with the Benjamini-Hochberg post hoc test correcting for multiple comparisons. The significance level was set to *p*_adjusted_ ≤ 0.05. Data visualization was carried out in R.

## 3. Results

### 3.1. Donor Characterization and Fecal Baseline Characteristics

Of eight recruited pediatric patients with CD, five subjects were female and three were male. The mean age was 13 ± 4 years. Subjects displayed widespread Bristol stool scale values ranging from 2 to 6 and a large variation in fecal dry weight (29 ± 14%). Fecal calprotectin levels (1899 ± 1700) were also heterogeneous, with values as low as 324 µg/g and as high as 5700 µg/g (Table 1).

**Figure 1:**
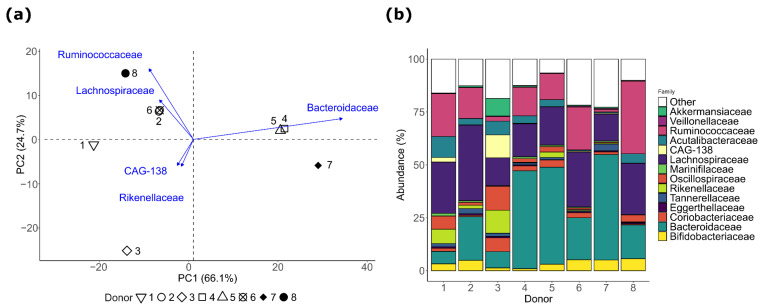
Microbial composition at family level of eight pIBD donors. Summary of relative microbial composition as (**a**) principal component plot and (**b**) bar plot.

To assess the interindividual variation, PCA was performed at the family level, revealing marked differences in microbial composition between pIBD donors, with two principal components explaining 90.8% of overall variation (Figure 1a). Donors 4, 5, and 7 were characterized by elevated *Bacteroidaceae* abundances, while the remaining donors were characterized by elevated levels of *Ruminococcaceae* (donor 8), *Lachnospiraceae* (donors 2, 6, and 8), and *Rikenellaceae* (donors 1 and 3; Figure 1a,b). 

### 3.2. HMOs and HMO Blends Exhibit Higher Acetogenic and Propiogenic Effects on pIBD Fecal Microbiota than FOS

**Figure 2:**
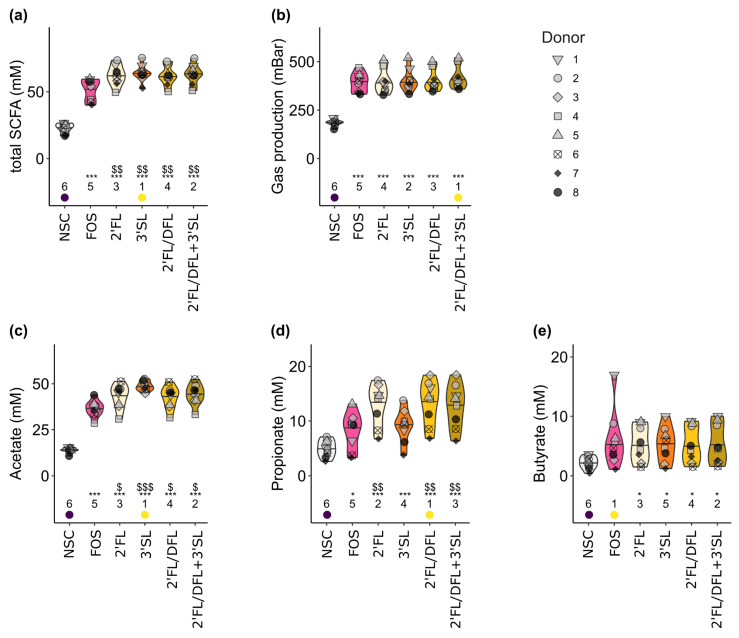
Impacts of HMOs and HMO blends on short-chain fatty acid (SCFA) profile and gas production of pIBD fecal microbiota. Violin plots of (**a**) total SCFAs, (**b**) gas production, (**c**) acetate, (**d**) propionate, and (**e**) butyrate levels. Plots depict mean and individual microbiota values for each donor (*n* = 8). The ranks of mean values per treatment are indicated at the bottom of the figure, with the lowest average being indicated in purple and the highest value in yellow. Significant effects between NSC and treatments are depicted with * (*p*_adjusted_ ≤ 0.05) and *** (*p*_adjusted_ ≤ 0.001). Significant effects between FOS and treatments are depicted with $ (*p*_adjusted_ ≤ 0.05), $$ (*p*_adjusted_ ≤ 0.01), and $$$ (*p*_adjusted_ ≤ 0.001).

To assess the effects of HMOs and FOS on the functionality of pIBD microbiota, SCFAs, BCFAs, pH, and gas production were quantified in fermentations of 24 h. HMO and FOS treatments significantly (*p*_adjusted_ ≤ 0.001) increased total SCFA levels when compared with the NSC (Figure 2a). In general, HMO containing treatments had a stronger stimulatory effect on total SCFA production than FOS (*p*_adjusted_ ≤ 0.01; Figure 2a). HMO and FOS treatments significantly (*p*_adjusted_ ≤ 0.001) increased gas production when compared with the NSC, while there was no significant difference between the FOS and HMO treatments (Figure 2b). 

Concurring with increased carbohydrate fermentation, milieu pH and proteolytic activity (BCFA levels) were significantly reduced (*p*_adjusted_ ≤ 0.05) in the HMO and FOS treatments when compared with the NSC. No significant difference in BCFA levels between the HMO and FOS treatments were observed, while 2′FL, 2′FL/DFL and 2′FL/DFL + 3′SL resulted in a significantly lower (*p*_adjusted_ ≤ 0.05) milieu pH than FOS (Table S1). HMO and FOS treatments significantly (*p*_adjusted_ ≤ 0.001) increased acetate levels when compared with the NSC. All HMO treatments exhibited a significantly higher acetogenic effect than FOS (Figure 2c). The FOS (*p*_adjusted_ ≤ 0.05) and HMO (*p*_adjusted_ ≤ 0.001) treatments significantly increased propionate concentrations when compared with the NSC, while all HMOs except 3′SL also significantly (*p*_adjusted_ ≤ 0.01) increased propionate when compared with FOS (Figure 2d). All HMO treatments significantly increased (*p*_adjusted_ ≤ 0.05) butyrate levels when compared with the NSC, while no significant effect was observed for the FOS treatment. No differences between the HMO and FOS treatments were observed (Figure 2e). No significant effects with regards to valerate levels were observed (Table S1).

### 3.3. No Differences in Overall Community Diversity between HMO- and FOS-Treated pIBD Fecal Microbiota

**Figure 3:**
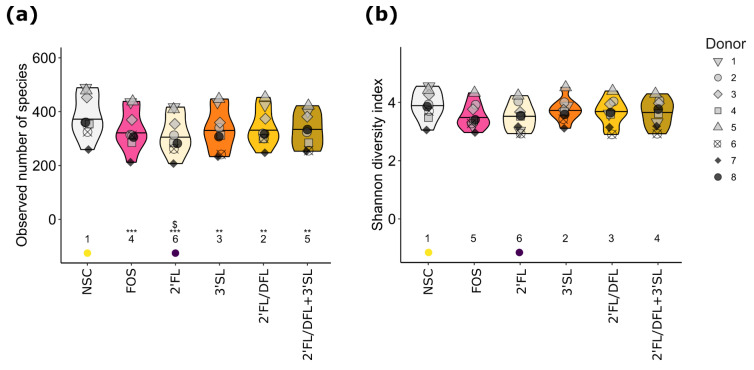
Impacts of HMOs and HMO blends on alpha diversity of pIBD fecal microbiota. Violin plots of (**a**) observed number of species (richness) and (**b**) Shannon diversity index (function of richness and evenness). Plots depict mean and individual microbiota values for each donor (*n* = 8). The ranks of mean values per treatment are indicated at the bottom of the figure, with the lowest average being indicated in purple and the highest value in yellow. Significant effects between NSC and treatments are depicted with ** (*p*_adjusted_ ≤ 0.01) and *** (*p*_adjusted_ ≤ 0.001). Significant effects between FOS and treatments are depicted with $ (*p*_adjusted_ ≤ 0.05).

To assess the effect of HMOs and FOS on alpha diversity (diversity within a community), the observed number of species (richness) and the Shannon diversity index (function of evenness and richness) were evaluated at 24 h. In general, HMO and FOS treatments significantly (*p*_adjusted_ ≤ 0.01) decreased richness when compared with the NSC (Figure 3a). Microbiota treated with 2′FL resulted in significantly (*p*_adjusted_ = 0.025) lower species richness than microbiota treated with FOS. Richness for 3′SL, 2′FL/DFL, and 2′FL/DFL + 3′SL treatments did not differ significantly (*p*_adjusted_ > 0.05) from FOS. There was, however, no significant difference in Shannon diversity between HMO and FOS treatments and the NSC (Figure 3b).

### 3.4. HMOs and HMO Blends Exhibit a Stronger Bifidogenic Effect on pIBD Fecal Microbiota than FOS

**Figure 4:**
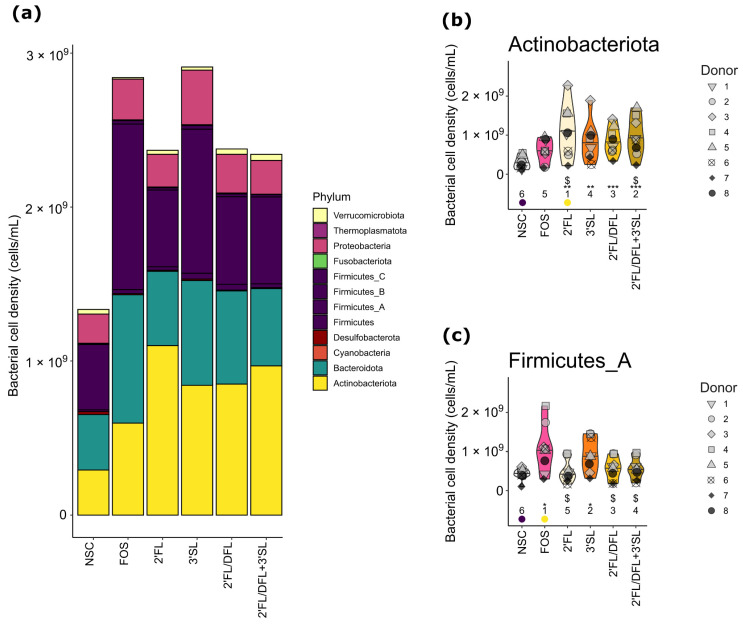
Impact of HMOs and HMO blends on composition, at phylum level, of pIBD fecal microbiota. (**a**) Bar plot of mean absolute abundance of different phyla. Violin plots of absolute abundance of (**b**) Actinobacteriota and (**c**) Firmicutes_A. Violin plots depict mean and individual microbiota values for each donor (*n* = 8). The ranks of mean values per treatment are indicated at the bottom of the figure, with the lowest average being indicated in purple and the highest value in yellow. Significant effects between NSC and treatments are depicted with * (*p*_adjusted_ ≤ 0.05), ** (*p*_adjusted_ ≤ 0.01), and *** (*p*_adjusted_ ≤ 0.001). Significant effects between FOS and treatments are depicted with $ (*p*_adjusted_ ≤ 0.05).

To evaluate the effects of HMOs and FOS on the fecal microbial composition, changes in the abundance of taxa at different taxonomic levels were assessed. The HMO and FOS treatments significantly increased (*p*_adjusted_ ≤ 0.01) total bacterial cell counts when compared with the NSC (Table S1; Figure 4a). Microbiota treated with 2′FL, 2′FL/DFL, and 2′FL/DFL + 3′SL exhibited significantly (*p*_adjusted_ ≤ 0.05) lower bacterial cell counts when compared with FOS, while no difference was observed between 3′SL and FOS (Table S1).

At the phylum level, HMO treatments significantly increased (*p*_adjusted_ ≤ 0.01) Actinobacteriota counts when compared with the NSC (Figure 4b). Moreover, 2′FL and 2′FL/DFL + 3′SL resulted in significantly higher (*p*_adjusted_ ≤ 0.05) Actinobacteriota counts when compared with FOS (Figure 4b). Microbiota treated with 3′SL and FOS had significantly higher (*p*_adjusted_ ≤ 0.05) Firmicutes_A counts when compared with the NSC (Figure 4c). Furthermore, FOS treatment significantly (*p*_adjusted_ ≤ 0.05) increased Firmicutes_A counts when compared with 2′FL, 2′FL/DFL, and 2′FL/DFL + 3′SL (Figure 4c). 

**Figure 5:**
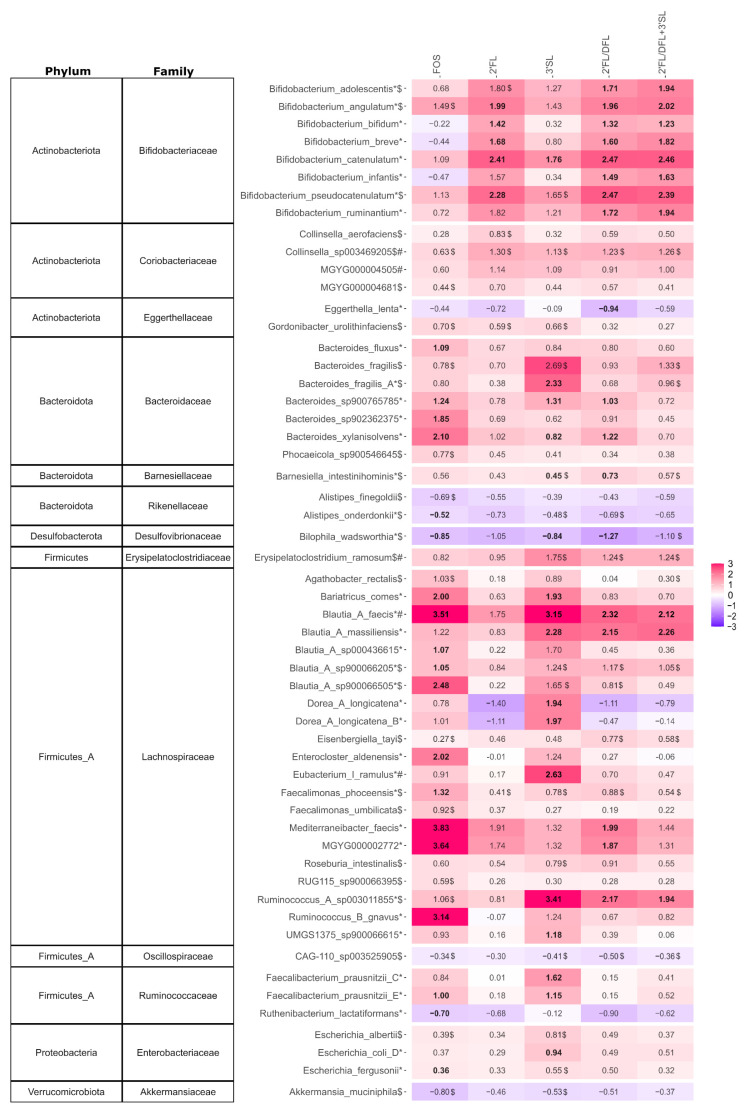
Impact of HMOs and HMO blends on the composition at species level of pIBD fecal microbiota. Heatmap of geometric mean of log_2_ fold changes against the NSC. Only species that were significantly (*p*_adjusted_ ≤ 0.05, indicated by *) or consistently (consistent but non-significant change in all donors (minimum four) where species are detected, indicated by $) affected by any treatment or being among the top 5 taxa explaining overall variation in the dataset based on PCA (indicated by #) were included. The analysis is based on the 150 most abundant species. Log_2_ fold changes that are significant are indicated in bold.

HMO and FOS treatment led to significant differences in various species when compared with the NSC (Figure 5). *Bifidobacterium angulatum*, *B. bifidum*, *B. breve*, *B. catenulatum*, and *B. pseudocatenulatum*, were significantly increased (*p*_adjusted_ ≤ 0.05) in 2′FL-treated microbiota when compared with the NSC. Additionally, 2′FL/DFL and 2′FL/DFL + 3′SL significantly (*p*_adjusted_ ≤ 0.05) enriched *B. adolescentis*, *B. infantis*, and *B. ruminantium*. In 3′SL-treated microbiota, only *B. catenulatum* was significantly (*p*_adjusted_ ≤ 0.05) enriched, exhibiting a lower log_2_ fold change (1.76) than observed for 2′FL, 2′FL/DFL, and 2′FL/DFL + 3′SL (>2; Figure 5). No *Bifidobacterium* species were significantly (*p*_adjusted_ > 0.05) enriched in the FOS-treated microbiota when compared with the NSC. In general, all HMO treatments exhibited a stronger bifidogenic effect when compared with FOS (Figure 5). *Bifidobacterium* species positively correlated with acetate levels for both HMO and FOS treatments, except for 2′FL/DFL + 3′SL (Figure S1).

The 2′FL and 2′FL/DFL+3′SL treatments did not significantly (*p*_adjusted_ > 0.05) affect *Bacteroides* species abundance when compared with the NSC, whereas 3′SL and 2′FL/DFL significantly (*p*_adjusted_ ≤ 0.05) increased abundance of three and two *Bacteroides* species, respectively. The 3′SL treatment mainly increased *B. fragilis* by 2.33 log_2_ fold changes when compared with the NSC. FOS more prominently enriched *Bacteroides* species, significantly (*p*_adjusted_ ≤ 0.05) increasing the levels of *B. fluxus*, *B. sp900765785*, *B. sp902362375*, and *B. xylanisolvens* in the range of 1.09 to 2.10 log_2_ fold changes when compared with the NSC (Figure 5). Thus, HMOs had weaker *Bacteroides*-promoting effects when compared with FOS.

The 3′SL, 2′FL/DFL, and 2′FL/DFL + 3′SL treatments exhibited a significant (*p*_adjusted_ ≤ 0.05) enrichment of *Blautia faecis* and *B. massiliensis* species, while no significant changes (*p*_adjusted_ > 0.05) in *Blautia* species abundances were observed for 2′FL when compared with the NSC. FOS significantly (*p*_adjusted_ ≤ 0.05) enriched *B. faecis*, *B. sp00436615*, *B. sp900066205*, and *B. sp900066505* when compared with the NSC (Figure 5). Thus, HMOs had a weaker effect on overall *Blautia* species abundance than FOS. *Blautia* species positively correlated with acetate and propionate for HMO and FOS treatments, except for 2′FL/DFL + 3′SL (Figure S1).

Treatments with 2′FL, 2′FL/DFL, and 2′FL/DFL + 3′SL had no significant (*p*_adjusted_ > 0.05) effects on *Faecalibacterium prausnitzii* species abundance when compared with the NSC. In contrast, 3′SL significantly (*p*_adjusted_ ≤ 0.05) enriched two different *F. prausnitzii* species in the range of 1.15–1.62 log_2_ fold changes. FOS significantly (*p*_adjusted_ ≤ 0.05) enriched one *F. prausnitzii* species by 1.00 log_2_ fold when compared with the NSC (Figure 5). Thus, 2′FL, 2′FL/DFL, and 2′FL/DFL + 3′SL had weaker effects on *F. prausnitzii* abundance when compared with FOS, while 3′SL had a stronger effect. 

## 4. Discussion

HMOs are a promising treatment and management strategy for IBD, given their anti-inflammatory properties, through direct effects on host immune and epithelial cells and modulation of the gut microbiota [14]. To date, there are, however, no insights on how different HMOs modulate the gut microbial community in pIBD patients. This study presents an in-depth analysis of the modulatory properties of 2′FL, DFL, 3′SL, and blends thereof compared to a NSC and FOS on pIBD microbiota. 

Amongst the fecal donors sourced for the present study, we observed strong interindividual variation in microbiota composition. This is in line with previous studies illustrating stratification of fecal microbiota based on composition [27,28] and suggests a good coverage of the versatile microbial composition observed in vivo by present donors. Treatment with 2′FL, 2′FL/DFL, and 2′FL/DFL + 3′SL exhibited a stronger propiogenic and acetogenic effect, and 3′SL alone had a stronger acetogenic effect compared with FOS. The significantly higher levels of propionate in 2′FL-treated microbiota when compared with FOS were previously not observed for healthy adult and pediatric microbiota [23], suggesting a distinct effect of HMOs (at least 2′FL) in pIBD patients. Indeed, previous research has shown altered SCFA profiles in pIBD patients. However, there were conflicting results between studies in terms of an increase or decrease of propionate and butyrate production (reviewed in [11]). SCFAs are essential in mediating the host inflammatory response by affecting innate and adaptive immunity and modulating gut epithelial cell wall integrity [29]. Thus, therapeutic strategies involving SCFAs have been proposed to mitigate inflammation in corresponding diseases [30]. Indeed, high acetate levels have recently been shown to mitigate inflammatory protein production and to have a beneficial effect on epithelial barrier integrity in an organoid model of UC patients [31]. The administration of HMOs to pIBD patients could thus beneficially alter the SCFA profile and aid in reestablishing epithelial barrier integrity and attenuating inflammation.

Although traditional prebiotics are well established to increase SCFA production, their application in IBD patients can be limited due to excessive bloating at higher doses, often reported with fructans such as inulin and FOS [32,33]. In contrast, HMOs have been shown to decrease bloating in patients with irritable bowel syndrome [34], suggesting that HMOs are a potential therapy for GI conditions. Our data support this notion by showing that HMO supplementation increased SCFA production beyond what was observed with the prebiotic FOS, yet kept gas production at similar levels. Thus, HMOs might be a viable alternative to traditional fructan-based prebiotics, limiting gas production while simultaneously enhancing SCFA concentrations.

An elevated microbial community richness is often associated with host health benefits [35]. Conversely, a decrease in microbial richness has been observed in pIBD patients [10]. Here, we show a general reduction of fecal microbial community richness with both FOS and HMO treatments. As previous studies have shown, a reduction in microbial richness is common for single fermentable substances in in vitro systems, given the lack of a variety of carbohydrate sources [36]. A reduction could also be attributed to an increasing cell density due to the addition of carbon sources (i.e., FOS or HMOs) and thus a decrease of the fraction of the community being sequenced [37].

Here, we showed that supplementation of pIBD microbiota with 2′FL-containing HMO blends markedly increased the abundance of *Bifidobacterium* species, including *B. infantis* and *B. bifidum,* while no significant effects of FOS on the *Bifidobacterium* community were observed. Many *Bifidobacterium* strains have long been established as probiotics with capabilities to shape the immune system and thus modulate inflammation [38]. As such, *B. infantis* species have been shown to exhibit anti-inflammatory effects in murine models of IBD and have been shown to contribute to an increased remission in UC patients [39,40]. Considering the depletion of *Bifidobacterium* species in pIBD patients [12], the present results suggest that HMOs are a promising solution to restore this community. We also found a positive correlation between *Bifidobacterium* species and acetate, suggesting a major contribution of these species to the observed acetogenic effects of 2′FL, 2′FL/DFL, and 3′SL. This observation aligns with the metabolic capabilities described for *Bifidobacterium* species [41]. However, the observed enrichment of *Bifidobacterium* species may not only exhibit beneficial effects on the host via metabolite production but also via surface polysaccharides. Cell surface β-glucans/galactans of *B. bifidum* have been shown to induce regulatory T cells, which ameliorated colitis in murine IBD models [8]. Even though we showed that 3′SL is not as efficient in stimulating *Bifidobacterium* species, we also found that 3′SL stimulates other potentially beneficial species, such as *Faecalibacterium prausnitzii*. Even more so, the stimulation of *F. prausnitzii* by 3′SL is higher than observed for FOS. A depletion of *F. prausnitzii* abundance in pIBD patients has been observed previously [10,12]. *F. prausnitzii* has been attributed with health benefits most likely mediated via butyrate and microbial anti-inflammatory molecule production [42]. It has been shown that *F. prausnitzii* grows on N-acetylneuraminic acid [43], one of the building blocks of 3′SL [14]. An enrichment of *F. prausnitzi* in ex vivo-cultivated healthy pediatric gut microbiota by 3′SL was also described previously [23], though at lower fold changes than observed in the present study.

Treatment with 2′FL did not enrich any *Blautia* species, while 3′SL, 2′FL/DFL, and 2′FL/DFL + 3′SL exhibited an enrichment of two *Blautia* species when compared to the NSC. However, 3′SL, 2′FL/DFL, and 2′FL/DFL + 3′SL showed an overall weaker effect on *Blautia* species abundance than FOS. Akin to the metabolic properties of *Blautia* taxa [41], we show a positive correlation with acetate and propionate levels for FOS, 2′FL, 3′SL, and 2′FL/DFL. *Blautia* taxa aid in maintaining mucus integrity via SCFA secretion [44] and have recently been described as having anti-inflammatory properties [45,46]. Thus, *Blautia* species have been suggested as novel probiotics [44]. Together with the notion that abundances of *Blautia* species have been reported to be decreased in pIBD patients [10,12], 3′SL, 2′FL/DFL, and 2′FL/DFL + 3′SL may counter dysbiosis and enrich bacteria with anti-inflammatory capabilities.

We show here that 3′SL significantly enriches *Bacteroides fragilis* abundance when compared with a NSC. However, 3′SL did not significantly enrich *B. fragilis* in healthy pediatric subjects [23], suggesting different responses of diseased and healthy microbiota. *B. fragilis* strains have been associated with beneficial effects. As such, they were shown to exhibit anti-inflammatory properties and alleviate colitis in a mouse IBD model, most likely via the secretion of polysaccharide A [47,48]. However, other studies do not support these health benefits [49].

The current study has several limitations. (i) Firstly, it must be noted that fecal samples only serve as a *proxy* of the dynamic environment and microbiota in the colon. Concurring with nutrient availability, water adsorption, and pH, the composition and functionality of gut microbes differ across the longitudinal (proximal to distal colon) and lateral (mucosa to lumen) axes of the colon [50]. However, a recent study has revealed the predictive nature of the SIFR^®^ technology for clinical gut microbiota research [25]. (ii) Secondly, here, the functionality of microbiota is evaluated via key fermentation parameters (i.e., SCFAs, BCFAs, pH, and gas production). However, the effect of HMOs on microbiota and host health is not limited to only these key parameters. As such, HMOs may also modulate other metabolites, such as tryptophan catabolite (i.e., indole-3-lactic acid and indole-3-propionate [23,51]) and secondary bile acid profiles [52,53]. These metabolites have been suggested to contribute to the amelioration of inflammatory processes [54,55]. (iii) Thirdly, we focus on HMO–microbe interactions. How HMOs and altered bacterial and metabolic profiles affect host cells in pIBD patients remains to be evaluated. Thus, the present results should be interpreted with caution, especially with regard to the management and treatment of pIBD. 

## 5. Conclusions

In conclusion, we show that HMOs beneficially alter the fecal microbial profile in pIBD, specifically enhancing the abundance of anti-inflammatory species and exhibiting an acetogenic and propiogenic effect in ex vivo conditions. These effects were stronger when compared with FOS. Further clinical studies are needed to fully evaluate the potential of HMOs as treatment and management strategies for pIBD.

## Figures and Tables

**Table 1:** Demographics and fecal baseline factors of pediatric IBD patients.

Donor	Sex	Age [Years]	Fecal Calprotectin [µg/g]	BSS	Fecal Dry Weight [%]
1	f	15	1300	4	41
2	m	17	1700	5	33
3	m	13	2680	2	51
4	f	11	324	2	34
5	f	9	1768	4	30
6	f	8	1100	6	15
7	m	18	622	5	9
8	f	16	5700	4	17

BSS: Bristol stool scale, f: female, m: male.

## Data Availability

The data generated during the current study are available from the corresponding author upon reasonable request.

## Supplementary Materials

The following supporting information can be downloaded at: https://www.mdpi.com/article/10.3390/microorganisms12101977/s1, Figure S1: Regularized canonical correlation analysis (rCCA) between bacterial composition and metabolites.; Table S1: Fermentation parameters, including pH, valerate, and branched-chain fatty acid (BCFA) concentrations for the non-substrate control (NSC) and all treatments.
