# Supplementary material for: Bifidogenic Effect of Human Milk Oligosaccharides on Pediatric IBD Fecal Microbiota
Source: Microorganisms. 2024 Sep 30;12(10):1977. doi: 10.3390/microorganisms12101977 (PMC11509818; doi:10.3390/microorganisms12101977)
Supplement: Supplementary file 1 [file microorganisms-12-01977-s001.zip › microorganisms-3171861-supplementary.pdf]

Supplementary File:

Table S1: Fermentation parameters including pH, valerate, and branched chain fatty acid (BCFA) concentrations for the non-substrate control (NSC) and all treatments. Values are given as mean of eight donors including standard deviation.

**Treatment**	**pH**	**Valerate [mM]**	**BCFA [mM]**	**Cell counts [log10(cells/mL)]**
NSC	6.63 ± 0.04	0.51 ± 0.38	1.12 ± 0.57	9.12 ± 0.07
FOS	6.11 ± 0.14[a]	0.35 ± 0.30	0.63 ± 0.30[a]	9.44 ± 0.11[a]
2'FL	5.92 ± 0.11[a,b]	0.31 ± 0.31	0.52 ± 0.37[a]	9.35 ± 0.14[a,b]
3'SL	6.14 ± 0.06[a]	0.31 ± 0.32	0.67 ± 0.31[a]	9.46 ± 0.07[a]
2'FL/DFL	5.93 ± 0.11[a,b]	0.32 ± 0.32	0.52 ± 0.35[a]	9.36 ± 0.14[a,b]
2'FL/DFL+3'SL	5.95 ± 0.12[a,b]	0.32 ± 0.32	0.52 ± 0.37[a]	9.35 ± 0.12[a,b]

a: significant effect ($p_{adjusted} \leq 0.05$) between NSC and treatments; b: significant effect ($p_{adjusted} \leq 0.05$) between FOS and HMO treatments; NSC: non-substrate control; FOS: fructooligosaccharide; 2'FL: 2'Fucosyllactose; 3'SL: 3'Sialyllactose; DFL: Difucosyllactose

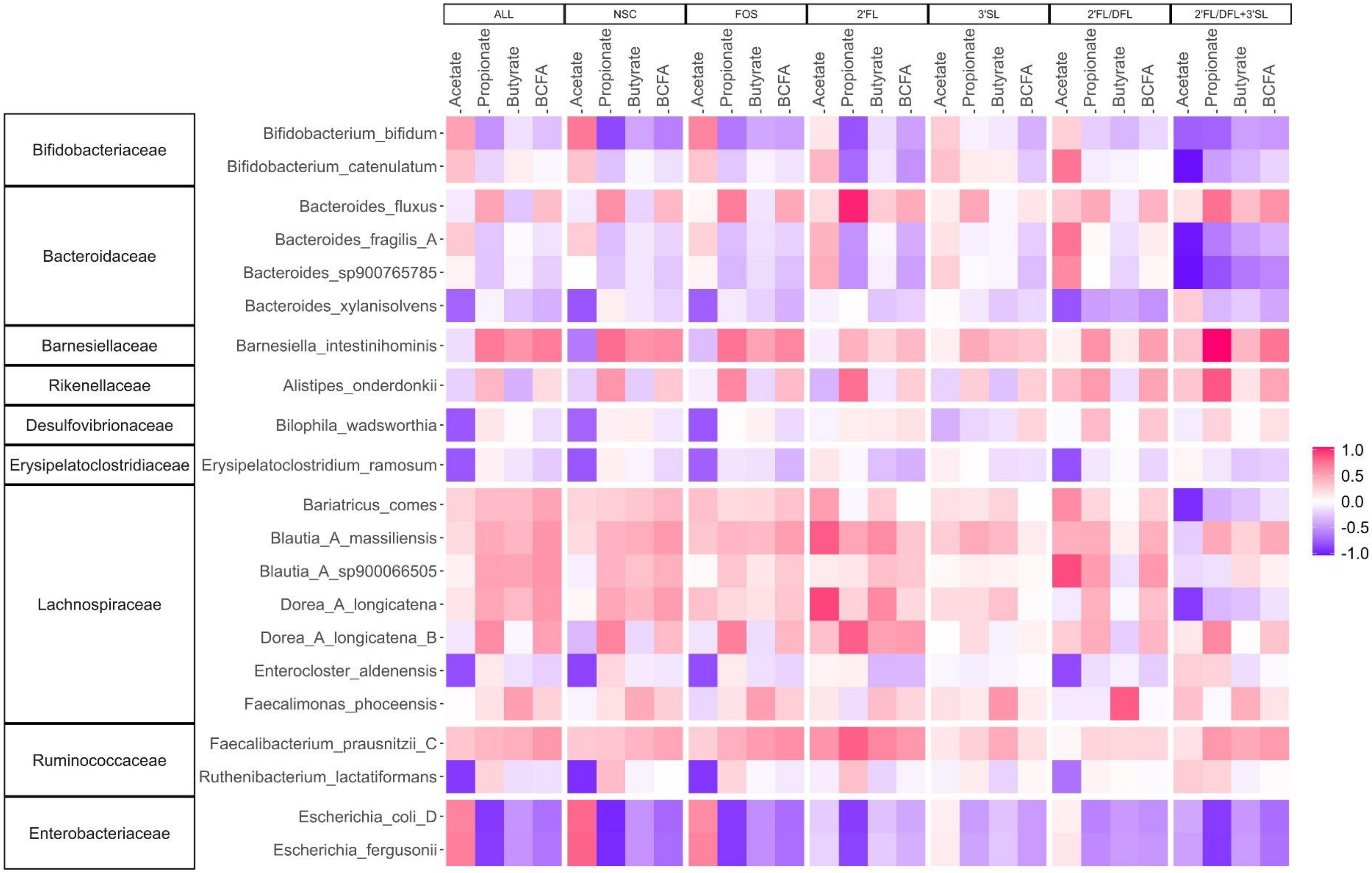

Figure S1: Regularized canonical correlation analysis (rCCA) between bacterial composition and metabolites. Composition is stated at species level including taxa that were significantly ($p_{\text{adjusted}} \leq 0.05$) or consistently affected by any treatment. Bacterial metabolites include acetate, propionate, butyrate and BCFA. Threshold was set to > 0.76.
